# Regional venous–arterial CO_2_ to arterial–venous O_2_ content difference ratio in experimental circulatory shock and hypoxia

**DOI:** 10.1186/s40635-020-00353-9

**Published:** 2020-10-29

**Authors:** Thiago Domingos Corrêa, Adriano José Pereira, Jukka Takala, Stephan Mathias Jakob

**Affiliations:** 1 Department of Intensive Care Medicine, Inselspital, Bern University Hospital, University of Bern, Bern, Switzerland; 2 Intensive Care Unit, Hospital Israelita Albert Einstein, Av. Albert Einstein, 627/701, 5th floor, São Paulo, 05651-901 Brazil; 3 Research Group, Hospital Municipal da Vila Santa Catarina, São Paulo, Brazil; 4 Postgraduate Program of Health Sciences, Federal University of Lavras, Lavras, Brazil

**Keywords:** Lactate, Lactate kinetics, Resuscitation, Oxygen consumption, Carbon dioxide, Septic shock, Multiple organ failure, Hypoxia

## Abstract

**Background:**

Venous–arterial carbon dioxide (CO_2_) to arterial–venous oxygen (O_2_) content difference ratio (Cv-aCO_2_/Ca-vO_2_) > 1 is supposed to be both sensitive and specific for anaerobic metabolism. What regional hemodynamic and metabolic parameters determine the ratio has not been clarified.

**Objectives:**

To address determinants of systemic and renal, spleen, gut and liver Cv-aCO_2_/Ca-vO_2_.

**Methods:**

Post hoc analysis of original data from published experimental studies aimed to address effects of different fluid resuscitation strategies on oxygen transport, lactate metabolism and organ dysfunction in fecal peritonitis and endotoxin infusion, and from animals in cardiac tamponade or hypoxic hypoxia. Systemic and regional hemodynamics, blood flow, lactate uptake, carbon dioxide and oxygen-derived variables were determined. Generalized estimating equations (GEE) were fit to assess contributors to systemic and regional Cv-aCO_2_/Ca-vO_2_.

**Results:**

Median (range) of pooled systemic Cv-aCO_2_/Ca-vO_2_ in 64 pigs was 1.02 (0.02 to 3.84). While parameters reflecting regional lactate exchange were variably associated with the respective regional Cv-aCO_2_/Ca-vO_2_ ratios, only regional ratios were independently correlated with systemic ratio: renal Cv-aCO_2_ /Ca-vO_2_ (*β* = 0.148, 95% CI 0.062 to 0.234; *p* = 0.001), spleen Cv-aCO_2_/Ca-vO_2_ (*β* = 0.065, 95% CI 0.002 to 0.127; *p* = 0.042), gut Cv-aCO_2_/Ca-vO_2_ (*β* = 0.117, 95% CI 0.025 to 0.209; *p* = 0.013), liver Cv-aCO_2_/Ca-vO_2_ (*β* = − 0.159, 95% CI − 0.297 to − 0.022; *p* = 0.023), hepatosplanchnic Cv-aCO_2_/Ca-vO_2_ (*β* = 0.495, 95% CI 0.205 to 0.786; *p* = 0.001).

**Conclusion:**

In a mixed set of animals in different shock forms or during hypoxic injury, hepatosplanchnic Cv-aCO_2_/Ca-vO_2_ ratio had the strongest independent association with systemic Cv-aCO_2_/Ca-vO_2_, while no independent association was demonstrated for lactate or hemodynamic variables.

## Background

Circulatory shock can be defined as a condition where tissue oxygen delivery does not meet the metabolic needs [1]. Inadequate oxygen delivery may also occur with normal perfusion when arterial oxygen content is critically reduced, e.g., in severe respiratory failure or at high altitude. In septic shock, both global perfusion and arterial oxygen content can be normal, but the metabolic needs may still be insufficiently met as a result of oxygen extraction and/or utilization abnormalities [2]. In all of these conditions, regional blood flow may be redistributed as a consequence of central (e.g., sympathetic nervous system) and/or peripheral (e.g., nitric oxide, shear stress) adaptive mechanisms [3].

In clinical practice, lactate is commonly used as an indicator of adequacy of tissue perfusion [4]. The recently released sepsis-3 definition includes increased blood lactate concentration as a criterion for the diagnosis of septic shock [1]. However, lactate concentration can also increase as a result of impaired ability of the liver to extract lactate [5] or as a consequence of increased aerobic lactate production, e.g., when epinephrine is used to stabilize hemodynamics [6].

When tissue oxygen delivery falls under a critical threshold, oxygen consumption and CO_2_ production from aerobic metabolism decrease [7]. Since some CO_2_ is produced from anaerobic metabolism, CO_2_ production falls less than O_2_ consumption and therefore, their ratio increases [7]. The combination of venous–arterial CO_2_ to arterial–venous O_2_ content difference ratio (Cv-aCO_2_/Ca-vO_2_) with arterial lactate levels has been reported to correlate with Sequential Organ Failure Assessment (SOFA) score and mortality [8].

Venous–arterial CO_2_ partial pressure to arterial–venous O_2_ content difference ratio (Pv-aCO_2_/Ca-vO_2_) is a relatively easily obtainable surrogate of the Cv-aCO_2_/Ca-vO_2_: pO_2_ and PCO_2_, oxygen saturation (SO_2_) and hemoglobin (Hb) are widely available in all modern blood gas analyzers, while CO_2_ content is not easy to measure or calculate. Pv-aCO_2_/Ca-vO_2_ has been used to predict systemic hyperlactatemia in critically ill patients [9]. However, it has been shown that decreased blood flow, rather than tissue hypoxia/dysoxia is the main determinant of increased venous–arterial CO_2_ gradient (Pv-aCO_2_) [10]. Furthermore, Pv-aCO_2_ may vary with different values of oxygen saturation due to Haldane effect, and due to changes in dissociation curves if pH, hemoglobin or temperature is changing. Accordingly, Pv-aCO_2_ may vary at constant total CO_2_ content [11].

What circulatory or metabolic parameters are best associated with systemic (Cv-aCO_2_/Ca-vO_2_) remains unclear. The aim of the present study was to address potential determinants of systemic and regional (renal, spleen, gut and liver) Cv-aCO_2_/Ca-vO_2_ in a mixed set of animals in different shock forms or during hypoxic injury. We hypothesized that parameters of hepatosplanchnic perfusion and metabolism are best predicting changes in systemic Cv-aCO_2_/Ca-vO_2_.

## Methods

We used original data from previously published studies [5, 12, 13]. They were performed in accordance with the National Institutes of Health guidelines for the care and use of experimental animals and with the approval of the Animal Care Committee of the Canton of Bern, Switzerland.

The first study (COHORT-1) was designed to address the impact of two different fluid resuscitation strategies (moderate-volume and high-volume replacement) on mortality, sepsis-associated organ dysfunction and mitochondrial function in animals submitted to either fecal peritonitis or endotoxin infusion [12].

The second study (COHORT-2) was performed to evaluate oxygen transport, lactate handling and mitochondrial function in animals challenged with fecal peritonitis, cardiac tamponade or hypoxic hypoxia [5, 13].

In both studies, animals were followed until 24 h after randomization or until death, if earlier. After 24 h, animals were euthanized with an overdose of potassium chloride [5, 12, 13]. The full study protocols of original studies can be found elsewhere [5, 12, 13].

### Surgical preparation

With animals in supine position, a midline laparotomy was performed and abdominal cavity exposed [5, 12, 13]. Catheters for pressure monitoring and blood sampling were inserted into the carotid, hepatic and pulmonary arteries and into jugular, hepatic, portal, renal, mesenteric and splenic veins [5, 12, 13]. A large bore catheter for fluid administration was inserted into the femoral vein. Ultrasound Doppler flow probes (Transonic^®^ Systems Inc., Ithaca, NY, USA) were placed around the carotid, superior mesenteric, hepatic, splenic and renal arteries, celiac trunk and around the portal vein [5, 12, 13]. A drainage catheter was inserted into the urinary bladder. Finally, two large bore drains were inserted via both flanks of the animals. The surgical procedure was followed by a 12-h period of hemodynamic stabilization [5, 12, 13].

#### Experimental protocol COHORT-1

After a 12-h period of hemodynamic stabilization, animals were randomized into six groups (*n* = 8 each, total 48 pigs) as follows: fecal peritonitis, endotoxin, or controls, each with either moderate-volume fluid resuscitation (10 ml/kg/h of Ringer's lactate) or high [15 ml/kg/h Ringer's lactate + 5 ml/kg/h hydroxyethyl starch (HES) 130/04, 6% (Voluven^®^, Fresenius, Stans, Switzerland)] for 24 h or until death, if earlier [12].

Fecal peritonitis was induced by peritoneal instillation through a peritoneal drainage tube of 1 g per kg body weight of autologous feces dissolved in 200 ml glucose 5% solution [12]. In the other groups, the same amount of sterile glucose solution was instilled. The intra-peritoneal drains were maintained clamped during the following 6 h [12]. In the endotoxin groups, endotoxin (lipopolysaccharide from Escherichia coli 0111:B4, 20 mg/l in 5% dextrose; Sigma^®^, Steinheim, Germany) was infused into the right atrium [12].

#### Experimental protocol COHORT-2

After 12-h period of hemodynamic stabilization, animals were randomized for a 24-h experiments into four groups (*n* = 8 each; total 32 pigs) as follow: fecal peritonitis, cardiac tamponade, hypoxic hypoxia, and controls [5, 13]. Following the 3R principles, COHORT-1 and 2 studies shared the control (*n* = 8) and fecal peritonitis groups (*n* = 8).

Fecal peritonitis was induced by instilling 1 g per kg body weight of autologous feces, dissolved in warmed glucose solution, in the abdominal cavity. In the other groups, the same amount of sterile glucose solution was instilled. The intra-peritoneal drains were clamped during the first 6 h [5, 13]. Cardiac tamponade was induced by filling the pericardial sac with HES 130/04, 6% (Voluven^®^, Fresenius Kabi, Bad Homburg, Germany) through the catheter positioned during the operation, aiming at a cardiac output of 60 ml/kg/min at 6 h, 50 ml/kg/min at 12 h, 40 ml/kg/min at 18 h, and 30 ml/kg/min at 24 h, respectively [13]. Hypoxic hypoxia was induced by reducing the fraction of inspired oxygen (FiO_2_) to 21% at 6 h, 18% at 12 h, 17% at 18 h, and 16% at 24 h [13]. When FiO_2_ was reduced below 21%, nitrogen was added to the gas mix. All pigs received Ringer’s lactate solution (10 ml/kg/h) during the experiment [13].

### Blood measurements

Blood samples for the measurement of hemoglobin (OSM3, Pig module, Radiometer, Copenhagen, Denmark), blood gases (ABL 520, Radiometer, Copenhagen, Denmark), and lactate (YSI 2300 Stat Plus, Yellow Springs Instruments, CA, USA) were taken at baseline and after 3, 6, 12, 18 and 24 h from pulmonary and carotid arteries, and from hepatic, portal, renal, mesenteric and splenic veins [5, 12, 13]. Blood sampling was also performed before 24 h if the animals exhibited a persistent decrease in mean arterial pressure (MAP) below 50 mmHg; these samples were considered as end values [5, 12, 13].

### Calculations

Systemic oxygen delivery (DO_2_) and consumption (VO_2_) and CO_2_ variables were calculated according the standard formulas as follows [5, 12, 13]:

- DO_2_ = CaO_2_ × CO
- VO_2_ = (CaO_2_ − CvO_2_) × CO
- O_2_ER = (CaO_2_ − CvO_2_)/CaO_2_
- CaO_2_ = (Hb × SaO_2_ × 1.34) × (PaO_2_ × 0.0031)
- CvO_2_ = (Hb x SvO_2_ × 1.34) × (PvO_2_ × 0.0031)
- Da-vO_2_ = CaO_2_ − CvO_2_
- Pv-aCO_2_ = PvCO_2_ − PaCO_2_,

where CO represents cardiac output expressed in ml/kg/min, Hb represents hemoglobin expressed in g/dl, O_2_ER represents the oxygen extraction ratio, CaO_2_ and CvO_2_ represent the arterial and venous O_2_ content expressed in ml/dl and PaCO_2_ and PvCO_2_ represent, respectively, arterial and venous CO_2_ partial pressures expressed in mmHg. CO_2_ contents were calculated according to the Douglas formula [11]:

- Plasma CO_2_ solubility (*S*) = 0.0307 + [0.00057 × (37 – *T*)] + [0.00002 × (37 − *T*)^2^], were temperature (*T*) is expressed as °C.
- pK’ = 6.086 + [0.042 × (7.4 − pH)] + [(38 − *T*) × {0.00472 + [0.00139 × (7.4 − pH)]}], were (pK’) is the apparent dissociation constant of carbonic acid, and (pH) is the potential of hydrogen (acidity).
- Plasma CO_2_ content (plasma CCO_2_, ml/dl) = 2.226 × *S* × plasma PCO_2_ × [1 + (10^pH–pK’^)], where (*S*) is the solubility coefficient for CO_2_.
- Blood CO_2_ content (Blood CCO_2_, ml/dl) = Plasma CCO_2_ × 1 − {(0.0289 × Hb) ÷ [3.352 − (0.456 × SO_2_)] × (8.142 − pH)]}, where SO_2_ is the oxygen saturation in the blood.

Time course of blood lactate levels (lactate disappearance) was defined as change in blood lactate levels (%) during a 6-h period [14]. Blood lactate exchange were calculated as follows [5, 14]:

- Hepatic lactate uptake (µmol/kg/min) = hepatic lactate influx − hepatic lactate efflux.
- Hepatic lactate influx (µmol/kg/min) = (portal venous lactate × portal vein blood flow) + (arterial lactate × hepatic arterial blood flow).
- Hepatic lactate efflux (µmol/kg/min) = hepatic venous lactate × (portal venous blood flow + hepatic arterial blood flow).
- Other regional lactate exchanges (renal, gut and spleen): regional lactate influx–regional lactate efflux.
- Whole body venous efflux (µmol/kg/min): cardiac output × mixed venous lactate concentration.

### Statistical analysis

All data are presented as *n*/*n* total (%) or median values with range. Normality was tested by the Kolmogorov–Smirnov test. Relationship between arterial lactate and base excess was assessed in the whole cohort with simple linear regression and Pearson correlation coefficient. Cv-aCO_2_/Ca-vO_2_ were compared between the different regions with Kruskal–Wallis test followed by pairwise comparisons with Mann–Whitney *U* test.

To account for longitudinal and correlated response variables, regression analysis based on generalized estimating equations (GEE) were fit to assess contributors to regional (renal, spleen, gut and liver) and systemic Cv-aCO_2_/Ca-vO_2_. Contributors to regional (renal, spleen, gut and liver) Cv-aCO_2_/Ca-vO_2_ included into the models were perfusion pressure [mean arterial blood pressure (MAP) minus central venous pressure (CVP)], venous hemoglobin, pH, pCO_2_, pO_2_, base excess and lactate, regional blood flow, regional DO_2_, VO_2_, and O_2_ER, regional lactate uptake and lactate gradient (regional venous lactate–arterial lactate). All regional (renal, spleen, gut and liver) predictors showing a *p* < 0.05 were entered into a final model aiming to address contributors to systemic Cv-aCO_2_/Ca-vO_2_. Unstandardized coefficients (*β*) along with their 95% confidence interval (95% CI) were reported.

To perform a sensitivity analyses, the model was re-assessed including only observations in which systemic Cv-aCO_2_/Ca-vO_2_ was higher than 1.0, which indicates anaerobic metabolism [7]. To address potential group effects, the experimental group (control group, fecal peritonitis, endotoxin infusion, cardiac tamponade and hypoxic hypoxia) was introduced as a predictor into the final models.

The SPSS™ (IBM™ Statistical Package for the Social Science version 26.0) were used for statistical analyses and GraphPad Prism version 7.0 (GraphPad Software, California, USA) was used for graph plotting.

## Results

Sixty-four domestic pigs (48 from cohort-1, and 16 from cohort-2) of both sexes [weight: 41.5 (35.0–48.9) kg, median (range)] were included in this study. Overall mortality was 40.6% (26/64 animals), with a median survival time of 24 (7–24) h. Mortality accordingly to experimental study groups is presented in Additional file 1: Table S1.

### Systemic hemodynamics, oxygen transport and lactate kinetics

Systemic hemodynamics, oxygen transport, acid–base balance and lactate kinetics from each study group have been previously reported [5, 12, 13]. The median of achieved values at different time points for the whole studied cohort are displayed in Table 1.

**Table 1:** Systemic hemodynamics, arterial lactate levels and whole-body venous lactate efflux

Parameters	BL	3 h	6 h	12 h	18 h	End
Heart rate (beats/min)	106 (74–178)	124 (83–214)	142 (81–222)	162 (86–222)	157 (77–200)	149 (74–206)
MAP (mm Hg)	70 (46–117)	78 (40–123)	73 (45–131)	75 (38–116)	74 (33–109)	68 (31–135)
MPAP (mm Hg)	17 (11–29)	23 (11–55)	21 (12–37)	24 (14–38)	23 (15–38)	25 (14–43)
CVP (mm Hg)	4 (0–10)	5 (1–12)	6 (2–12)	7 (1–15)	7 (2–14)	8 (2–18)
Cardiac output (ml/kg/min)	78 (50–120)	88 (38–154)	93 (53–146)	90 (47–202)	101 (40–167)	105 (38–301)
SVRI (mm Hg l/kg/min)	836 (501–1347)	869 (346–2274)	808 (408–1972)	776 (410–1249)	684 (151–1471)	598 (198–1315)
SvO_2_ (%)	50 (33–67)	55 (18–70)	55 (8–75)	55 (19–69)	57 (16–70)	53 (6–73)
DO_2_ (ml/min)	384 (224–654)	421 (200–991)	447 (236–781)	407 (190–816)	441 (161–700)	440 (150–980)
VO_2_ (ml/min)	181 (96–274)	178 (108–310)	184 (109–311)	181 (115–393)	190 (105–282)	197 (105–363)
O_2_ER	0.48 (0.32–0.66)	0.42 (0.27–0.74)	0.44 (0.23–0.91)	0.43 (0.26–0.75)	0.41 (0.28–0.84)	0.43 (0.24–0.94)
Lactate (mmol/l)	0.6 (0.4–2.3)	0.8 (0.2–2.7)	0.7 (0.4–2.2)	0.8 (0.5–3.6)	0.7 (0.4–2.0)	1.0 (0.4–7.0)
Lactate disappearance (%)			− 16 (− 286 to 43)	1 (− 339 to 54)	− 5 (− 184 to 49)	− 7 (− 308 to 34)
Whole-body venous lactate efflux (µmol/kg/min)	47.5 (28.9–139.0)	68.8 (22.9–222.6)	71.8 (27.8–301.4)	70.8 (30.0–183.4)	79.9 (30.4–237.8)	91.9 (29.3–513.8)

Values represent median and range. Lactate disappearance (%): initial lactate − final lactate/initial lactate × 100. Whole body venous efflux (μmol/kg/min): cardiac output × mixed venous lactate concentration

*BL* baseline, *End* of the experiment (at 24 h of resuscitation or before death if earlier), *MAP* mean arterial blood pressure, *MPAP* mean pulmonary artery pressure, *CVP* central venous pressure, *SVRI* systemic vascular resistance index, *SvO*_2_ mixed venous oxygen saturation, *DO*_2_ systemic oxygen delivery, *VO*_2_ systemic oxygen consumption and *O*_2_*ER* systemic oxygen extraction

Briefly, animals submitted to fecal peritonitis became hypotensive while cardiac output, systemic DO_2_ and arterial lactate levels increased. Endotoxin infusion resulted increased cardiac output and mean pulmonary artery pressure (MPAP) while systemic VO_2_ and O_2_ER remained stable. Cardiac tamponade animals exhibited systemic arterial hypotension and decreased DO_2_ while systemic O_2_ER and arterial lactate levels increased. Hypoxic hypoxia resulted in increased cardiac output while systemic DO_2_ and O_2_ER remained unchanged [5, 12, 13].

### Systemic carbon dioxide and oxygen variables

Acid–base balance from each study group have been previously reported [5, 12, 13]. Base excess inversely correlated with arterial lactate levels (*r* = − 0.65, *p* < 0.001; Additional file 1: Figure S1). The median of achieved values at different time points for the whole studied cohort are displayed in Table 2.

**Table 2:** Arterial blood gas analysis, hemoglobin and systemic carbon dioxide and oxygen variables

Parameters	BL	3 h	6 h	12 h	18 h	End
pH	7.48 (7.41–7.53)	7.46 (7.31–7.52)	7.45 (7.29–7.54)	7.44 (7.25–7.58)	7.44 (7.32–7.56)	7.41 (7.18–7.59)
Bicarbonate (mmol/l)	27.2 (23.9–29.6)	26.3 (22.0–29.9)	26.2 (21.6–30.8)	26.6 (19.8–30.9)	26.9 (21.8–31.8)	26.9 (13.9–33.5)
Base excess (mmol/l)	3.9 (0.1–6.5)	2.8 (− 2.8 to 6.6)	2.8 (− 2.8 to 7.4)	3.0 (− 4.9 to 8.2)	3.2 (− 2.2 to 8.5)	2.6 (− 9.0 to 8.7)
PaCO_2_ (mm Hg)	37.7 (33.1–41.8)	38.0 (29.7–50.7)	38.6 (31.3–51.5)	39.3 (33.3–53.1)	38.6 (34.0–56.7)	41.1 (17.7–80.3)
PaO_2_ (mm Hg)	137 (81–177)	119 (49–214)	121 (43–172)	118 (34–183)	127 (38–203)	110 (44–218)
SpO_2_ (%)	97 (90–100)	96 (69–100)	96 (55–99)	96 (55–98)	96 (50–97)	95 (46–98)
Arterial hemoglobin (g/dl)	8.9 (6.3–11.2)	9.2 (7.0–13.8)	9.4 (6.4–14.2)	9.1 (6.6–13.5)	8.4 (6.6–13.7)	8.6 (5.2–13.0)
CaCO_2_ (ml/dl)	56.5 (49.5–61.4)	54.6 (44.5–62.4)	54.9 (44.7–63.5)	55.4 (43.5–64.0)	57.0 (45.4–66.4)	56.0 (28.4–73.5)
CvCO_2_ (ml/dl)	62.3 (47.2–67.2)	60.0 (51.4–68.3)	59.5 (51.4–69.2)	61.1 (52.0–67.9)	62.0 (52.5–69.4)	61.9 (43.9–75.1)
Cv-aCO_2_ (ml/dl)	5.7 (1.9–10.0)	5.5 (0.1–9.6)	5.4 (1.0–10.8)	5.5 (1.8–10.5)	5.5 (2.3–9.7)	5.1 (1.6–19.3)
Pv-aCO_2_ (mmHg)	8.4 (0.9–11.7)	8.4 (3.1–15.1)	7.8 (1.7–14.2)	8.9 (2.9–16.7)	8.5 (0.5–18.6)	9.4 (4.6–64.4)
CaO_2_ (ml/dl)	12.1 (8.7–14.9)	12.3 (8.2–17.5)	12.5 (7.0–18.4)	11.8 (6.6–18.3)	11.2 (5.3–18.2)	10.7 (5.3–17.1)
CvO_2_ (ml/dl)	6.3 (2.9–8.9)	6.9 (2.1–11.6)	7.1 (1.0–13.5)	6.7 (2.7–11.0)	6.8 (1.5–11.2)	6.1 (0.8–12.8)
Ca-vO_2_ (ml/l)	5.6 (4.2–8.1)	5.7 (3.5–8.1)	5.2 (2.8–9.2)	5.2 (2.6–9.0)	4.4 (3.0–9.6)	4.6 (2.9–11.8)
Cv-aCO_2_/Ca-vO_2_	1.0 (0.4–1.7)	1.0 (0.0–1.6)	1.0 (0.3–1.8)	1.0 (0.5–1.6)	1.1 (0.5–2.0)	1.0 (0.3–3.8)
Pv-aCO_2_/Ca-vO_2_ (mmHg ml O_2_/dl)	1.5 (0.1–2.2)	1.5 (0.8–2.8)	1.5 (0.4–2.1)	1.7 (1.0–2.6)	1.9 (0.1–2.5)	2.0 (1.1–6.2)

Values represent median and range. BL = baseline, End = end of experiment after 24 h of randomization or before death if earlier. PaO_2_ = arterial oxygen partial pressure, PaCO_2_ = arterial carbon dioxide partial pressure, SpO_2_ = peripheral oxygen saturation, CaCO_2_ = arterial CO_2_ content, CvCO_2_ = venous CO_2_ content, Cv-aCO_2_ = venous–arterial CO_2_ content difference, Pv-aCO_2_ = venous-to-arterial carbon dioxide difference, CaO_2_ = arterial O_2_ content, CvO_2_ = venous O_2_ content, Ca-vO_2_ = arterial–venous O_2_ content difference, Cv-aCO_2_/Ca-vO_2_ = venous–arterial CO_2_ to arterial–venous O_2_ content difference ratio, Pv-aCO_2_/Ca-vO_2_ = ratio between venous-to-arterial carbon dioxide difference and arterial–venous oxygen content difference

Median of pooled systemic Cv-aCO_2_/Ca-vO_2_ was 1.02 (0.02 to 3.84). During the experimental period, median Cv-aCO_2_ and Ca-vO_2_ decreased while their ratio remained unchanged (Table 2).

**Fig. 1:**
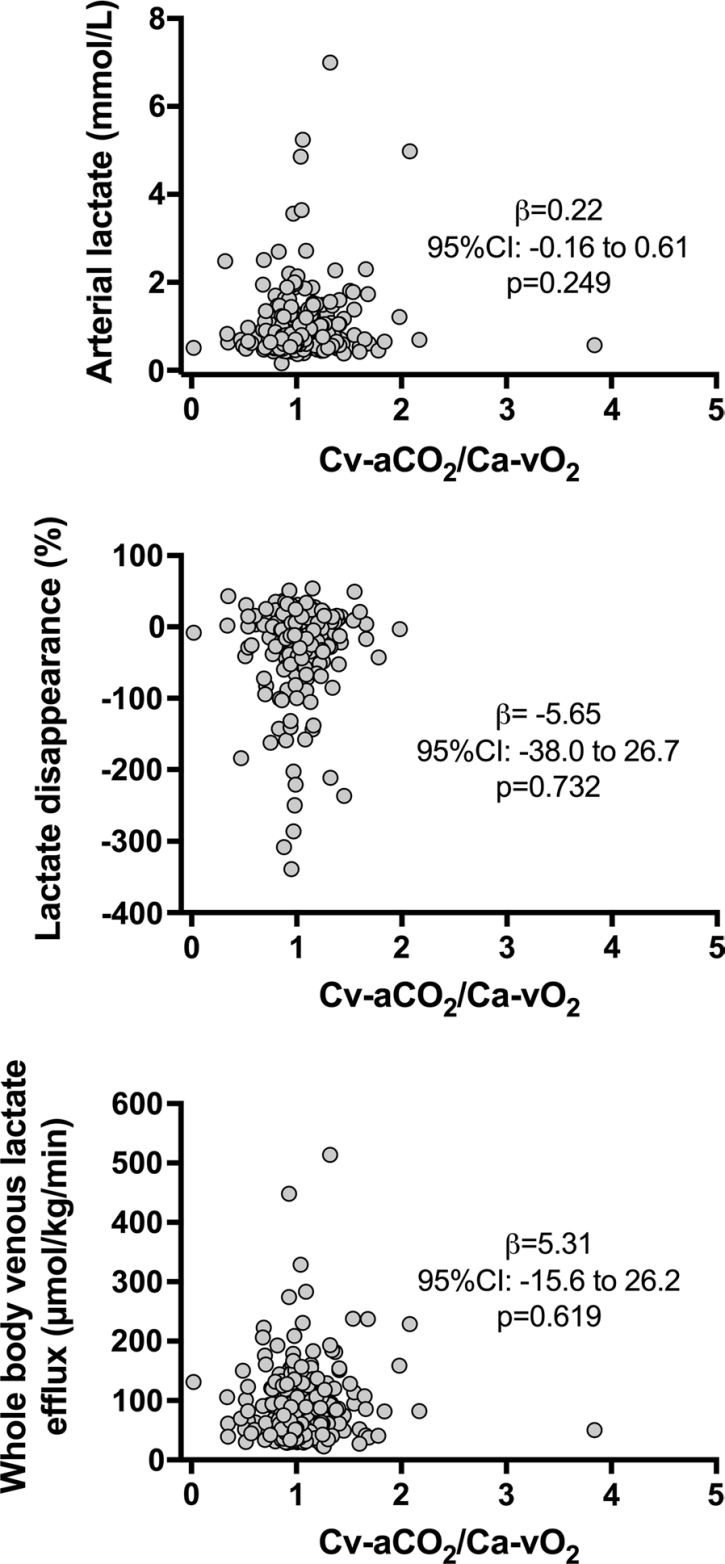
Scatter plot between systemic venous–arterial CO_2_ to arterial–venous O_2_ content difference ratio (Cv-aCO_2_/Ca-vO_2_) and arterial lactate, lactate disappearance and whole-body venous lactate efflux. *β* = unstandardized coefficient, CI = confidence interval, Cv-aCO_2_/Ca-vO_2_ = venous–arterial CO_2_ to arterial–venous O_2_ content difference ratio, whole-body venous efflux = cardiac output × mixed venous lactate concentration, lactate disappearance (%) = initial lactate − final lactate/initial lactate × 100

Systemic Cv-aCO_2_/Ca-vO_2_ was neither correlated with arterial lactate (*β* = 0.22, 95% CI − 0.16 to 0.61; *p* = 0.249), lactate disappearance (*β* = − 5.65, 95% CI − 38.0 to 26.7; *p* = 0.732) nor with whole-body venous lactate efflux (*β* = 5.31, 95% CI − 15.6 to 26.2; *p* = 0.619) (Fig. 1).

### Regional blood flow, lactate uptake and Cv-aCO_2_/Ca-vO_2_

Median total hepatic blood flow, total hepatosplanchnic blood flow, superior mesenteric artery blood flow and spleen artery blood flow increased during the experimental period, with maximal values observed at the end of study. Renal artery blood flow remained stable during experimental period (Table 3). Hepatic lactate uptake and hepatosplanchnic lactate uptake increased during the study period and were markedly positive (lactate extraction). Renal, mesenteric and splenic lactate uptake, which were all slightly negative (lactate release), remained stable (Table 3).

**Table 3:** Regional blood flow, lactate uptake and venous–arterial CO_2_ to arterial–venous O_2_ content difference ratio

Parameters	BL	3 h	6 h	12 h	18 h	End
Total hepatic blood flow (ml/kg/min)	22.3 (9.7–37.8)	22.5 (12.0–35.2)	24.0 (12.0–41.0)	25.3 (8.7–49.1)	28.3 (9.4–50.3)	27.3 (8.4–55.7)
Total hepatosplanchnic blood flow (ml/kg/min)	23.7 (14.7–42.9)	23.5 (9.9–39.4)	25.4 (15.1–40.7)	27.6 (16.5–50.4)	31.4 (14.0–55.0)	31.1 (9.7–69.2)
Hepatic lactate uptake (µmol/kg/min)	8.5 (2.1–26.5)	10.0 (3.5–30.1)	9.8 (− 2.4 to 30.0)	10.9 (0.8–22.4)	9.8 (− 0.5 to 23.4)	10.8 (− 22.8–25.4)
Hepatosplanchnic lactate uptake (µmol/kg/min)	6.8 (0.0–60.1)	7.4 (− 7.2 to 43.9)	8.2 (− 3.8 to 27.6)	8.2 (− 0.7 to 25.1)	8.2 (− 2.3 to 33.7)	7.2 (− 32.3–31.6)
Lactate hepatic vein − hepatic artery (µmol/l)	− 0.3 (− 1.6 to 0.0)	− 0.3 (− 1.8 to 0.4)	− 0.3 (− 1.3 to 0.1)	− 0.3 (− 1.0 to 0.0)	− 0.3 (− 1.6 to 0.2)	− 0.3 (− 1.3 to 1.4)
Lactate hepatic vein − portal vein (µmol/l)	− 0.4 (− 0.6 to -0.1)	− 0.5 (− 2.1 to -0.1)	− 0.4 (− 1.0 to 0.1)	− 0.4 (− 1.2 to 0.0)	− 0.4 (− 1.6 to 0.0)	− 0.4 (− 0.9 to 1.7)
Hepatic Cv-aCO_2_/Ca-vO_2_	0.7 (0.0–1.6)	0.8 (0.1–3.2)	0.8 (0.1–2.9)	0.8 (0.2–2.2)	0.9 (0.5–2.1)	0.9 (0.2–6.9)
Hepatosplanchnic Cv-aCO_2_/Ca-vO_2_	1.0 (0.4–1.5)	1.0 (0.4–2.3)	1.0 (0.7–2.1)	1.0 (0.5–1.5)	1.0 (2.2–1.5)	1.0 (0.2–3.4)
Renal artery blood flow (ml/kg/min)	5.2 (1.4–9.5)	4.8 (1.1–9.3)	4.7 (2.0–12.5)	5.4 (1.7–10.7)	5.4 (1.8–10.4)	4.9 (0.8–11.8)
Renal lactate uptake (µmol/kg/min)	0.0 (− 3.9 to 8.5)	− 0.2 (− 5.1 to 6.9)	− 0.6 (− 5.4 to 1.2)	− 0.4 (− 5.9 to 3.0)	− 0.7 (− 5.2 to 3.4)	− 0.2 (− 5.7 to 6.3)
Renal lactate gradient (µmol/l)	0.0 (− 1.4 to 1.4)	0.0 (− 1.2 to 1.0)	0.1 (− 0.3 to 1.0)	0.1 (− 0.8 to 1.8)	0.2 (− 0.4 to 1.0)	0.1 (− 0.9 to 1.4)
Renal Cv-aCO_2_/Ca-vO_2_	1.2 (0.4–2.8)	1.0 (0.4–2.3)	1.1 (0.1–2.2)	1.2 (0.1–3.2)	1.1 (0.5–2.5)	1.1 (0.3–4.7)
Renal Pv-aCO_2_/Ca-vO_2_ (mmHg ml O_2_/dl)	1.4 (0.1–3.1)	1.4 (0.0–3.3)	1.3 (0.2–2.2)	1.7 (0.5–6.0)	1.7 (0.7–3.1)	1.7 (0.7–6.2)
Superior mesenteric artery blood flow (ml/kg/min)	15.5 (9.4–35.1)	15.9 (6.9–26.8)	16.7 (8.1–31.9)	17.5 (10.7–39.9)	18.3 (8.9–43.9)	18.8 (7.1–47.8)
Gut lactate uptake (µmol/kg/min)	− 1.7 (− 12.9–40.4)	− 2.0 (− 11.8–20.8)	− 2.0 (− 22.8–11.0)	− 2.1 (− 11.3–9.5)	− 2.5 (− 12.7–4.5)	− 2.6 (− 12.7–23.2)
Gut lactate gradient (µmol/l)	0.1 (− 1.2 to 0.8)	0.1 (− 1.2 to 0.8)	0.1 (− 0.6 to 1.3)	0.1 (− 0.5 to 0.6)	0.1 (− 0.3 to 0.5)	0.1 (− 1.9 to 0.8)
Gut Cv-aCO_2_/Ca-vO_2_	1.3 (0.5–3.9)	1.2 (0.3–2.8)	1.3 (0.5–3.0)	1.2 (0.1–2.1)	1.3 (0.4–2.0)	1.2 (0.4–5.0)
Gut Pv-aCO_2_/Ca-vO_2_ (mmHg ml O_2_/dl)	2.2 (0.2–6.8)	2.2 (0.7–4.2)	2.5 (1.1–4.0)	2.5 (1.5–4.5)	2.8 (1.5–4.2)	3.0 (1.1–8.4)
Spleen artery blood flow (ml/kg/min)	1.0 (0.2–3.3)	1.0 (0.4–4.6)	1.3 (0.2–4.9)	1.2 (0.2–5.2)	1.3 (0.2–6.6)	1.3 (0.1–8.2)
Spleen lactate uptake (µmol/kg/min)	− 0.1 (− 2.1 to 1.6)	− 0.3 (− 1.6 to 0.4)	− 0.3 (− 1.7 to 0.4)	− 0.2 (− 1.1 to 0.6)	− 0.2 (− 1.4 to 0.4)	− 0.3 (− 1.8 to 1.8)
Spleen lactate gradient (µmol/l)	0.1 (− 1.0 to 1.1)	0.2 (− 0.8 to 1.0)	0.2 (− 0.4 to 1.1)	0.2 (− 0.4 to 1.1)	0.2 (− 0.3 to 0.8)	0.2 (− 0.9 to 2.6)
Spleen Cv-aCO_2_/Ca-vO_2_	1.3 (0.4–3.2)	1.1 (0.3–2.9)	1.2 (0.2–3.1)	1.3 (0.0–5.6)	1.3 (0.3–3.9)	1.1 (0.5–6.0)
Spleen Pv-aCO_2_/Ca-vO_2_ (mmHg ml O_2_/dl)	2.2 (0.8–3.2)	2.3 (1.1–6.8)	2.4 (0.1–4.0)	2.4 (1.3–7.5)	2.6 (0.3–5.3)	2.9 (0.3–8.4)

Values represent median and range. BL = baseline, End = end of the experiment (at 24 h of resuscitation or before death if earlier). Regional blood flow was expressed as ml/kg/min. Total hepatic blood flow = hepatic arterial blood flow + portal vein blood flow, total hepatosplanchnic blood flow = celiac trunk blood flow + superior mesenteric artery blood flow, hepatic lactate uptake = hepatic lactate influx − hepatic lactate efflux, hepatosplanchnic lactate uptake = (arterial lactate − hepatic venous lactate) * total hepatic blood flow, Cv-aCO_2_/Ca-vO_2_ = venous–arterial CO_2_ to arterial–venous O_2_ content difference ratio, regional lactate uptake (renal, gut and spleen) = regional lactate influx − regional lactate efflux, regional lactate gradient (renal, gut and spleen) = regional venous lactate − arterial lactate, Pv-aCO_2_/Ca-vO_2_ = ratio between venous-to-arterial carbon dioxide difference and arterial–venous oxygen content difference

Median (range) of pooled hepatic, hepatosplanchnic, renal, mesenteric and spleen Cv-aCO_2_/Ca-vO_2_ were, respectively, 0.82 (0.0–6.9), 1.0 (0.2–3.4), 1.1 (0.1–4.7), 1.2 (0.1–5.0) and 1.2 (0.0–6.0) (Additional file 1: Fig. S2). Values for the whole cohort at different time points are displayed in Table 3, and separate values for the individual experimental groups in Additional file 1: Fig. S3.

Contributors to regional (renal, spleen, gut and liver) Cv-aCO_2_/Ca-vO_2_ for the whole cohort are presented in Additional file 1: Tables S2–S5). Contributors to regional (renal, spleen, gut and liver) Cv-aCO_2_ /Ca-vO_2_ for time points which systemic Cv-aCO_2_/Ca-vO_2_ > 1.0 only are presented in Additional file 1: Tables S6–S9.

### Contributors to systemic Cv-aCO_2_/Ca-vO_2_

Renal Cv-aCO_2_/Ca-vO_2_ (*β* = 0.148, 95% CI 0.062 to 0.234; *p* = 0.001), spleen Cv-aCO_2_/Ca-vO_2_ (*β* = 0.065, 95% CI 0.002 to 0.127; *p* = 0.042), gut Cv-aCO_2_/Ca-vO_2_ (*β* = 0.117, 95% CI 0.025 to 0.209; *p* = 0.013), liver Cv-aCO_2_/Ca-vO_2_ (*β* = − 0.159, 95% CI − 0.297 to − 0.022; *p* = 0.023) and hepatosplanchnic Cv-aCO_2_/Ca-vO_2_ (*β* = 0.495, 95% CI 0.205 to 0.786; *p* = 0.001)— but none of the other parameters—were independently correlated with systemic Cv-aCO_2_/Ca-vO_2_ (Table 4).

**Table 4:** Contributors to systemic venous–arterial CO_2_ to arterial–venous O_2_ content difference ratio (Cv-aCO_2_/Ca-vO_2_) (*n* = 255)

Parameters	*β*	95% CI	*p* value
Renal Cv-aCO_2_/Ca-vO_2_	0.148	0.062 to 0.234	0.001
Spleen Cv-aCO_2_/Ca-vO_2_	0.065	0.002 to 0.127	0.042
Gut Cv-aCO_2_/Ca-vO_2_	0.117	0.025 to 0.209	0.013
Liver Cv-aCO_2_/Ca-vO_2_	− 0.159	− 0.297 to − 0.022	0.023
Hepatosplanchnic Cv-aCO_2_/Ca-vO_2_	0.495	0.205 to 0.786	0.001
MAP-CVP (mmHg)	− 0.001	− 0.002 to 0.001	0.364
Base excess kidney vein (mmol/l)	0.001	− 0.012 to 0.014	0.873
Renal O_2_ER	− 0.025	− 0.271 to 0.221	0.843
Lactate kidney vein (mmol/l)	− 0.043	− 0.171 to 0.085	0.510
Renal lactate gradient (mmol/l)	0.104	− 0.036 to 0.245	0.144
Spleen lactate gradient (mmol/l)	0.037	− 0.051 to 0.124	0.411
Gut O_2_ER	0.089	− 0.164 to 0.342	0.489
Lactate mesenteric vein (mmol/l)	0.043	− 0.074 to 0.160	0.474
pO_2_ hepatic vein (mmHg)	0.002	− 0.005 to 0.009	0.563
Total hepatic blood flow (ml/kg/min)	− 0.001	− 0.009 to 0.006	0.729
Hepatic O_2_ER	0.132	− 0.174 to 0.438	0.397
Hepatic lactate uptake (µmol/kg/min)	0.000	− 0.007 to 0.007	0.969
Hepatosplanchnic lactate uptake (µmol/kg/min)	0.007	0.000 to 0.015	0.059
Lactate hepatic vein − hepatic artery (µmol/l)	0.075	− 0.122 to 0.271	0.456

*β* = unstandardized coefficient, CI = confidence interval, Cv-aCO_2_/Ca-vO_2_ = venous–arterial CO_2_ to arterial–venous O_2_ content difference ratio, MAP = mean arterial blood pressure, CVP = central venous pressure, O_2_ER = oxygen extraction, renal lactate gradient = lactate kidney vein − lactate arterial, spleen lactate gradient = lactate spleen vein − lactate arterial, pO_2_ = oxygen partial pressure, total hepatic blood flow = hepatic arterial blood flow + portal vein blood flow, hepatic lactate uptake = hepatic lactate influx − hepatic lactate efflux, hepatosplanchnic lactate uptake = (arterial lactate − hepatic venous lactate) * total hepatic blood flow

#### Additional analysis

When only systemic Cv-aCO_2_/Ca-vO_2_ > 1.0 were included in the analysis, only renal Cv-aCO_2_/Ca-vO_2_ (*β* = 0.099, 95% CI 0.039 to 0.159; *p* = 0.001), Gut Cv-aCO_2_/Ca-vO_2_ (*β* = 0.120, 95% CI 0.015 to 0.225; *p* = 0.025), liver Cv-aCO_2_/Ca-vO_2_ (*β* = − 0.323, 95% CI − 0.508 to − 0.138; *p* = 0.001) and hepatosplanchnic Cv-aCO_2_/Ca-vO_2_ (*β* = 0.641, 95% CI 0.274 to 1.008; *p* = 0.001) remained independently correlated with systemic Cv-aCO_2_/Ca-vO_2_ (Additional file 1: Table S10).

The inclusion of experimental group (fecal peritonitis, endotoxin infusion, cardiac tamponade and hypoxic hypoxia) as a predictor into the final models assessing contributors to Cv-aCO_2_/Ca-vO_2_ did not affect the results (Additional file 1: Tables S11 and S12).

## Discussion

We found that variables indicating regional lactate transport and oxygen extraction ratios correlated with regional but not systemic Cv-aCO_2_/Ca-vO_2_ ratios. The best predictor of systemic Cv-aCO_2_/Ca-vO_2_ ratio was the hepatosplanchnic Cv-aCO_2_/Ca-vO_2_ ratio (*β* = 0.495), while the other regional ratios correlated also, albeit to a weaker extent. If only systemic Cv-aCO_2_/Ca-vO_2_ > 1 were included in the analysis, also renal and spleen lactate gradients correlated with systemic Cv-aCO_2_/Ca-vO_2_ ratios.

In patients with acute lung injury, septic or cardiogenic shock, a Pv-aCO_2_/Ca-vO_2_ > 1.4 predicted hyperlactatemia [9]. In contrast to this study, we aimed to predict Cv-aCO_2_/Ca-vO_2_ ratios from regional blood flows, and lactate and other metabolism related variables. Our findings may be explained by a high regional heterogeneity in terms of tolerance to anemic and hypoxic insults [15], and differences in the availability of adaptive mechanisms among various organs/systems [16]. Therefore, markers indicating tissue dysoxia in one organ may be diluted by those of other organs with more resources to adapt.

In our fluid-resuscitated experimental models, arterial lactate levels increased only moderately, and not in all animals. Despite this, we found overall associations between lactate-related parameters and Cv-aCO_2_/Ca-vO_2_ in all regions. Since the regional Cv-aCO_2_/Ca-vO_2_ determined systemic Cv-aCO_2_/Ca-vO_2_ to some extent, we suggest that increasing systemic Cv-aCO_2_/Ca-vO_2_ is useful to detect subtle regional metabolic alterations, even if values are smaller than 1.

Lactate and lactic acidosis are not exclusively related to anaerobic metabolism [17]. Interpretation of lactate in shock and hypoxia has evolved from being a marker of anaerobic metabolism, to represent part of an adaptive mechanism to provide an alternative substrate to vital organs such as kidney, brain and heart (lactate shuttle theory) [18]. Experimental data support the concept that respiratory quotient, expressed as the ratio between CO_2_ production (VCO_2_), and oxygen consumption (VO_2_) is a reliable marker of anaerobic threshold [19]. Our study found that neither arterial lactate, nor lactate disappearance, nor whole-body venous lactate efflux are associated with RQ (measured as Cv-aCO_2_/Ca-vO_2_), potentially suggesting that lactate may be, at least in part, associated to aerobic metabolism in the hypoxia and shock models assessed in this study. The slower lactate kinetics of lactate compared to blood gas variables may provide another explanation in some cases, as discussed by others [8]. However, our data demonstrate that lactate concentrations > 2 mmol/l were almost always associated with negative base excess values. Finally, Cv-aCO_2_ may not increase in states of tissue hypoxia when venous blood flow is high enough to wash out CO_2_ produced by the hypoxic cells [20].

The hepatosplanchnic region was the most important contributor to systemic Cv-aCO_2_/Ca-vO_2._ It could be argued that this is a consequence of the greater amount of tissue perfused in this compared to the other regions. On the other hand, we have demonstrated in humans that changes in hepatosplanchnic metabolism and blood flow dissociate from those of systemic blood flow and metabolism [21, 22]. Furthermore, one has to acknowledge that blood flow is not a component of Cv-aCO_2_/Ca-vO_2_ values—the main drivers are pCO_2_, Hb and SO_2_. Therefore, higher rates of CO_2_ production related to O_2_ consumption in the hepatosplanchnic compared to other regions could have accounted for the high contribution of hepatosplanchnic to systemic Cv-aCO_2_/Ca-vO_2_. However, we found that Cv-aCO_2_/Ca-vO_2_ ratios were lowest in the hepatosplanchnic region. Nevertheless, in low flow and/or high metabolic circumstances, portal venous oxygen content and therefore hepatic oxygen delivery will decrease over-proportionally, also because portal flow represents around 2/3 of total liver perfusion—this renders the region, especially the liver, at risk for inadequate oxygen supply [23].

In terms of determinants of systemic Cv-aCO_2_/Ca-vO_2_, regional veno-arterial lactate gradients were only significantly associated when Cv-aCO_2_/Ca-vO_2_ was > 1 and specifically in kidney and spleen. Kidney is an important organ for lactate disposal, accounting for about 30% of its systemic turnover. In the kidney, lactate is used for gluconeogenesis and as a substrate for ATP production, similarly to brain and heart [18, 24]. On the other hand, spleen lactate metabolism is very poorly described. A recent paper about inter-organ metabolite exchange metabolomics mapped that spleen is one of the organs which the highest production of lactate in the body at rest [25]. In our study, spleen together with mesenteric region was also the organ with the highest Cv-aCO_2_/Ca-vO_2_ gradients, suggesting that this organ operated at a level closer to anaerobic condition than other organs.

Limitations of the present study include the retrospective nature (post hoc analysis of previous studies), and a pooling of several models. It seems obvious that evaluation of the parameters in each group separately would have resulted in more overt changes over time. The drawback of this approach is the relatively small n per group. The advantage of a pooled group is the large range of variables and a better simulation of a mixed ICU patient population. Conceptually, one would expect that higher lactate concentrations go together with higher Cv-aCO_2_/Ca-vO_2_ values, independently of group or time point, if both reflect tissue hypoxia. Furthermore, we did not find an effect of the model on the association between the various parameters with regional or systemic Cv-aCO_2_/Ca-vO_2_ ratios. Given the design of the original studies and the fixed time points of blood sampling, we could neither determine how fluid resuscitation in the sepsis models affected regional and systemic Cv-aCO_2_/Ca-vO_2_ ratios, nor the association of Cv-aCO_2_/Ca-vO_2_ ratios with signs of systemic inflammation and tissue injury. Therefore, we cannot provide mechanistic explanations for our findings—this should be done in further, prospective studies. Lastly, it can be argued, that hepatosplanchnic and splenic/mesenteric values are mathematically and physiologically coupled, as they share the arterial variables, and because the latter contribute to the former. By using a strict pre-analysis protocol to obtain blood and measure the parameters included in Cv-aCO_2_/Ca-vO_2_ values, and by measuring hemoglobin and oxygen saturation with a module specific for pig blood, we believe that measurement errors largely have been avoided. We acknowledge that, e.g., mesenteric Cv-aCO_2_/Ca-vO_2_ values contribute to hepatosplanchnic values—as all regional values contribute to systemic values. In the present study, we were interested in Cv-aCO_2_/Ca-vO_2_ values of both single organs (spleen, gut, kidney) and the whole hepatosplanchnic region—especially also because blood sampling from the liver vein is clinically feasible, whereas samples from portal and splenic veins can be obtained only intra-operatively. Conversely, strengths are the provision of data where data have been lacking so far, and the ethical commitment with the 3R principle (Replacement, Reduction and Refinement) on animal research.

The overall aim of this analysis was to improve our understanding about contributors to systemic Cv-aCO_2_/Ca-vO_2_ values, since the latter may offer a more specific indicator of anaerobic metabolism than lactate. While we found that only regional Cv-aCO_2_/Ca-vO_2_ contributed to the respective systemic values, contributors to regional Cv-aCO_2_/Ca-vO_2_ values differed in the regions (e.g., regional lactate gradient in the kidney, venous independent of arterial lactate in the gut; MAP-CVP in the hepatosplanchnic region but not in the kidney)—especially when regional Cv-aCO_2_/Ca-vO_2_ values were > 1. These data are preliminary and have not been prospectively studied. However, we believe they are important for hypothesis generation about how different regions can metabolically adapt to insults—and about potentially modifiable factors.

## Conclusion

In a mixed set of animals in different shock states or with hypoxic injury, regional variables representing local lactate transport and oxygen extraction ratios correlated with the respective regional Cv-aCO_2_/Ca-vO_2_ ratios. However, only regional Cv-aCO_2_/Ca-vO_2_ ratios were independently associated with systemic Cv-aCO_2_/Ca-vO_2_.

## Supplementary information

Additional file 1 (DOCX 750 kb) **Table S1.** Survival accordingly to study groups. Values represent *n*/*n* total (%).**Table S2.** Correlation between renal venous-arterial CO_2_ to arterial-venous O_2_ content difference ratio (Cv-aCO_2_/Ca-vO_2_) and systemic and regional hemodynamics, kidney vein hemoglobin and blood gas analysis, and lactate uptake (*n* = 313). **Table S3.** Correlation between spleen venous-arterial CO_2_ to arterial-venous O_2_ content difference ratio (Cv-aCO_2_/Ca-vO_2_) and systemic and regional hemodynamics, spleen vein hemoglobin and blood gas analysis, and lactate uptake (*n* = 294). **Table S4.** Correlation between gut venous-arterial CO_2_ to arterial-venous O_2_ content difference ratio (Cv-aCO_2_/Ca-vO_2_) and systemic and regional hemodynamics, mesenteric vein hemoglobin and blood gas analysis, and lactate uptake (*n *= 312). **Table S5.** Correlations between liver venous-arterial CO_2_ to arterial-venous O_2_ content difference ratio (Cv-aCO_2_/Ca-vO_2_) and systemic and regional hemodynamics, liver vein hemoglobin and blood gas analysis, and lactate uptake (*n* = 313). **Table S6.** Correlation between renal venous-arterial CO_2_ to arterial-venous O_2_ content difference ratio (Cv-aCO_2_/Ca-vO_2_) and systemic and regional hemodynamics, kidney vein hemoglobin and blood gas analysis, and lactate uptake (*n* = 170; including only systemic Cv-aCO_2_/Ca-vO_2_ > 1.0). **Table S7.** Correlation between spleen venous-arterial CO_2_ to arterial-venous O_2_ content difference ratio (Cv-aCO_2_/Ca-vO_2_) and systemic and regional hemodynamics, spleen vein hemoglobin and blood gas analysis, and lactate uptake (*n *= 162; including only systemic Cv-aCO_2_/Ca-vO_2_ > 1.0). **Table S8.** Correlation between gut venous-arterial CO_2_ to arterial-venous O_2_ content difference ratio (Cv-aCO_2_/Ca-vO_2_) and systemic and regional hemodynamics, mesenteric vein hemoglobin and blood gas analysis, and lactate uptake (*n* = 166; including only systemic Cv-aCO_2_/Ca-vO_2_ > 1.0). **Table S9.** Correlations between liver venous-arterial CO_2_ to arterial-venous O_2_ content difference ratio (Cv-aCO_2_/Ca-vO_2_) and systemic and regional hemodynamics, liver vein hemoglobin and blood gas analysis, and lactate uptake (n=171; including only systemic Cv-aCO_2_/Ca-vO_2_ > 1.0). **Table S10.** Contributors to systemic venous-arterial CO_2_ to arterial-venous O_2_ content difference ratio (Cv-aCO_2_/Ca-vO_2_) (*n* = 147; including only systemic Cv-aCO_2_/Ca-vO_2_ > 1.0). **Table S11.** Contributors to systemic venous-arterial CO_2_ to arterial-venous O_2_ content difference ratio (Cv-aCO_2_/Ca-vO_2_) after adjusting for experimental group (*n* = 255). **Table S12.** Contributors to systemic venous-arterial CO_2_ to arterial-venous O_2_ content difference ratio (Cv-aCO_2_/Ca-vO_2_) after adjusting for experimental group (*n* = 147; including only systemic Cv-aCO_2_/Ca-vO_2_ > 1.0). **Figure S1. **Linear regression and correlation between arterial lactate and base excess. **Figure S2.** Boxplot of pooled hepatic, hepatosplanchnic, renal, mesenteric and spleen venous-arterial CO_2_ to arterial-venous O_2_ content difference ratio (Cv-aCO_2_/Ca-vO_2_). **Figure S3.** Systemic and regional CO_2_ to arterial-venous O_2_ content difference ratio (Cv-aCO_2_/Ca-vO_2_) accordingly to experimental models.

## Data Availability

The datasets used and analyzed during the current study are available from the corresponding author on reasonable request.

## Abbreviations

*β*: Unstandardized coefficient
BL: Baseline
CI: Confidence interval
CO: Cardiac output
CaCO_2_: Arterial CO_2_ content
CvCO_2_: Venous CO_2_ content
Cv-aCO_2_: Venous–arterial CO_2_ content difference
CaO_2_: Arterial O_2_ content
CvO_2_: Venous O_2_ content
Ca-vO_2_: Arterial–venous O_2_ content difference
Cv-aCO_2_/Ca-vO_2_: Venous–arterial CO_2_ to arterial–venous O_2_ content difference ratio
CVP: Central venous pressure
DO_2_: Systemic oxygen delivery
End: End of the experiment (at 24 h of resuscitation or before death if earlier)
MAP: Mean arterial blood pressure
MPAP: Mean pulmonary artery pressure
O_2_ER: Systemic oxygen extraction
PaO_2_: Arterial oxygen partial pressure
PaCO_2_: Arterial carbon dioxide partial pressure
Pv-aCO_2_: Venous-to-arterial carbon dioxide difference
Pv-aCO_2_/Ca-vO_2_: Ratio between venous-to-arterial carbon dioxide difference and arterial–venous oxygen content difference
RQ: Respiratory quotient
SpO_2_: Peripheral oxygen saturation
SVRI: Systemic vascular resistance index
SvO_2_: Mixed venous oxygen saturation
VO_2_: Systemic oxygen consumption
