# Supplementary material for: Regional venous–arterial CO2 to arterial–venous O2 content difference ratio in experimental circulatory shock and hypoxia
Source: Intensive Care Med Exp. 2020 Oct 29;8:64. doi: 10.1186/s40635-020-00353-9 (PMC7596113; doi:10.1186/s40635-020-00353-9)
Supplement: Supplementary file 1 — Additional file 1 (DOCX 750 kb) Table S1. Survival accordingly to study groups. Values represent n/n total (%).Table S2. Correlation between renal venous-arterial CO2 to arterial-venous O2 content difference ratio (Cv-aCO2/Ca-vO2) and systemic and regional hemodynamics, kidney vein hemoglobin and blood gas analysis, and lactate uptake (n = 313). Table S3. Correlation between spleen venous-arterial CO2 to arterial-venous O2 content difference ratio (Cv-aCO2/Ca-vO2) and systemic and regional hemodynamics, spleen vein hemoglobin and blood gas analysis, and lactate uptake (n = 294). Table S4. Correlation between gut venous-arterial CO2 to arterial-venous O2 content difference ratio (Cv-aCO2/Ca-vO2) and systemic and regional hemodynamics, mesenteric vein hemoglobin and blood gas analysis, and lactate uptake (n = 312). Table S5. Correlations between liver venous-arterial CO2 to arterial-venous O2 content difference ratio (Cv-aCO2/Ca-vO2) and systemic and regional hemodynamics, liver vein hemoglobin and blood gas analysis, and lactate uptake (n = 313). Table S6. Correlation between renal venous-arterial CO2 to arterial-venous O2 content difference ratio (Cv-aCO2/Ca-vO2) and systemic and regional hemodynamics, kidney vein hemoglobin and blood gas analysis, and lactate uptake (n = 170; including only systemic Cv-aCO2/Ca-vO2 > 1.0). Table S7. Correlation between spleen venous-arterial CO2 to arterial-venous O2 content difference ratio (Cv-aCO2/Ca-vO2) and systemic and regional hemodynamics, spleen vein hemoglobin and blood gas analysis, and lactate uptake (n = 162; including only systemic Cv-aCO2/Ca-vO2 > 1.0). Table S8. Correlation between gut venous-arterial CO2 to arterial-venous O2 content difference ratio (Cv-aCO2/Ca-vO2) and systemic and regional hemodynamics, mesenteric vein hemoglobin and blood gas analysis, and lactate uptake (n = 166; including only systemic Cv-aCO2/Ca-vO2 > 1.0). Table S9. Correlations between liver venous-arterial CO2 to arterial-venous O2 content [file 40635_2020_353_MOESM1_ESM.docx]

**REGIONAL VENOUS-ARTERIAL CO_2_ TO ARTERIAL-VENOUS O_2_ CONTENT DIFFERENCE RATIO IN EXPERIMENTAL CIRCULATORY SHOCK AND HYPOXIA**

**Additional Information**

Authors: Thiago Domingos Corrêa, Adriano José Pereira, Jukka Takala and Stephan Mathias Jakob

**Table S1.** Survival accordingly to study groups. Values represent n/n total (%).

| **Experimental model** | **Mortality**  **(%)** | **Survival time (h)** |
| --- | --- | --- |
| Control low | 0/8 (0.0) | 24 (24-24) |
| Control high | 0/8 (0.0) | 24 (24-24) |
| Endotoxemia low | 1/8 (12.5) | 24 (9-24) |
| Endotoxemia high | 6/8 (75.0) | 19 (12-24) |
| Peritonitis low | 4/8 (50.0) | 24 (12-24) |
| Peritonitis high | 7/8 (87.5) | 18 (11-24) |
| Hypoxic hypoxia | 4/8 (50.0) | 23 (11-24) |
| Cardiac tamponade | 4/8 (50.0) | 24 (7-24) |
| Overall | 26/64 (40.6) | 24 (7-24) |

**Table S2.** Correlation between renal venous-arterial CO_2_ to arterial-venous O_2_ content difference ratio (Cv-aCO_2_/Ca-vO_2_) and systemic and regional hemodynamics, kidney vein hemoglobin and blood gas analysis, and lactate uptake (n=313).

| **Parameters** | **β** | **95%CI** | **p value** |
| --- | --- | --- | --- |
| MAP-CVP (mmHg) | -0.001 | 0.005 to 0.003 | 0.735 |
| Hemoglobin kidney vein (g/dl) | -0.002 | -0.060 to 0.056 | 0.945 |
| pH kidney vein | -7.065 | -15.569 to 1.440 | 0.104 |
| pCO_2_ kidney vein (mmHg) | -0.032 | -0.084 to 0.020 | 0.226 |
| pO_2_ kidney vein (mmHg) | -0.003 | -0.024 to 0.018 | 0.776 |
| Base excess kidney vein (mmol/L) | 0.156 | 0.038 to 0.275 | 0.010 |
| Renal artery blood flow (mL/kg/min) | -0.006 | -0.086 to 0.074 | 0.883 |
| Renal DO_2_ (ml/kg/min) | -0.329 | -1.011 to 0.354 | 0.345 |
| Renal VO_2_ (ml/kg/min) | 0.480 | -1.099 to 2.059 | 0.551 |
| Renal O_2_ER | -2.062 | -4.116 to -0.009 | 0.049 |
| Lactate kidney vein (mmol/L) | 0.241 | 0.103 to 0.379 | 0.001 |
| Kidney lactate uptake (µmol/kg/min) | 0.012 | -0.047 to 0.071 | 0.687 |
| Lactate gradient (mmol/L) | -0.436 | -0.744 to -0.128 | 0.005 |

**β** = unstandardized coefficient, CI = confidence interval, MAP = mean arterial blood pressure, CVP = central venous pressure, pCO_2_ = carbon dioxide partial pressure, pO_2_ = oxygen partial pressure, DO_2_ = oxygen delivery, VO_2_ = oxygen consumption, O_2_ER = oxygen extraction, lactate gradient = lactate kidney vein - lactate arterial.

**Table S3.** Correlation between spleen venous-arterial CO_2_ to arterial-venous O_2_ content difference ratio (Cv-aCO_2_/Ca-vO_2_) and systemic and regional hemodynamics, spleen vein hemoglobin and blood gas analysis, and lactate uptake (n=294).

| **Parameters** | **β** | **95%CI** | **p value** |
| --- | --- | --- | --- |
| MAP-CVP (mmHg) | -0.004 | -0.009 to 0.001 | 0.135 |
| Hemoglobin spleen vein (g/dl) | 0.002 | -0.061 to 0.064 | 0.962 |
| pH spleen vein | 12.304 | -2.410 to 27.019 | 0.101 |
| pCO_2_ spleen vein (mmHg) | 0.089 | -0.001 to 0.178 | 0.053 |
| pO_2_ spleen vein (mmHg) | 0.001 | -0.013 to 0.014 | 0.913 |
| Base excess spleen vein (mmol/L) | -0.119 | -0.329 to 0.092 | 0.268 |
| Spleen artery blood flow (mL/kg/min) | 0.076 | -0.286 to 0.438 | 0.681 |
| Spleen DO_2_ (ml/kg/min) | 0.170 | -2.595 to 2.935 | 0.904 |
| Spleen VO_2_ (ml/kg/min) | -4.145 | -9.118 to 0.828 | 0.102 |
| Spleen O_2_ER | -0.531 | -1.700 to 0.639 | 0.374 |
| Lactate spleen vein (mmol/L) | 0.104 | -0.039 to 0.248 | 0.153 |
| Spleen lactate uptake (µmol/kg/min) | -0.043 | -0.488 to 0.402 | 0.850 |
| Lactate gradient (mmol/L) | -0.635 | -1.150 to -0.121 | 0.016 |

**β** = unstandardized coefficient, CI = confidence interval, MAP = mean arterial blood pressure, CVP = central venous pressure, pCO_2_ = carbon dioxide partial pressure, pO_2_ = oxygen partial pressure, DO_2_ = oxygen delivery, VO_2_ = oxygen consumption, O_2_ER = oxygen extraction, lactate gradient = lactate spleen vein - lactate arterial.

**Table S4.** Correlation between gut venous-arterial CO_2_ to arterial-venous O_2_ content difference ratio (Cv-aCO_2_/Ca-vO_2_) and systemic and regional hemodynamics, mesenteric vein hemoglobin and blood gas analysis, and lactate uptake (n=312).

| **Parameters** | **β** | **95%CI** | **p value** |
| --- | --- | --- | --- |
| MAP-CVP (mmHg) | -0.004 | -0.007 to -0.001 | 0.008 |
| Hemoglobin mesenteric vein (g/dl) | -0.026 | -0.083 to 0.030 | 0.363 |
| pH mesenteric vein | 6.261 | -2.522 to 14.774 | 0.149 |
| pCO_2_ mesenteric vein (mmHg) | 0.044 | -0.009 to 0.097 | 0.101 |
| pO_2_ mesenteric vein (mmHg) | -0.012 | -0.027 to 0.004 | 0.141 |
| Base excess mesenteric vein (mmol/L) | -0.029 | -0.148 to 0.090 | 0.637 |
| Mesenteric artery blood flow (mL/kg/min) | -0.014 | -0.046 to 0.019 | 0.416 |
| Gut DO_2_ (ml/kg/min) | 0.061 | -0.334 to 0.455 | 0.763 |
| Gut VO_2_ (ml/kg/min) | 0.461 | -0.145 to 1.067 | 0.136 |
| Gut O_2_ER | -2.421 | -4.258 to -0.584 | 0.010 |
| Lactate mesenteric vein (mmol/L) | 0.198 | 0.093 to 0.304 | <0.001 |
| Gut lactate uptake (µmol/kg/min) | 0.014 | -0.004 to 0.032 | 0.136 |
| Lactate gradient* (mmol/L) | -0.082 | -1.481 to 0.317 | 0.687 |

**β** = unstandardized coefficient, CI = confidence interval, MAP = mean arterial blood pressure, CVP = central venous pressure, pCO_2_ = carbon dioxide partial pressure, pO_2_ = oxygen partial pressure, DO_2_ = oxygen delivery, VO_2_ = oxygen consumption, O_2_ER = oxygen extraction, lactate gradient = lactate mesenteric vein - lactate arterial.

**Table S5.** Correlations between liver venous-arterial CO_2_ to arterial-venous O_2_ content difference ratio (Cv-aCO_2_/Ca-vO_2_) and systemic and regional hemodynamics, liver vein hemoglobin and blood gas analysis, and lactate uptake (n=313).

| **Parameters** | **β** | **95%CI** | **p value** |
| --- | --- | --- | --- |
| MAP-CVP (mmHg) | -0.002 | -0.005 to -0.001 | 0.187 |
| Hemoglobin hepatic vein (g/dl) | -0.053 | -0.126 to 0.020 | 0.153 |
| pH hepatic vein | 8.295 | -3.255 to 19.846 | 0.159 |
| pCO_2_ hepatic vein (mmHg) | 0.069 | -0.002 to 0.141 | 0.057 |
| pO_2_ hepatic vein (mmHg) | -0.041 | -0.077 to -0.004 | 0.028 |
| Base excess hepatic vein (mmol/L) | -0.106 | -0.258 to 0.046 | 0.173 |
| Total hepatic blood flow (mL/kg/min) | -0.031 | -0.056 to -0.005 | 0.017 |
| Total hepatosplanchnic blood flow (mL/kg/min) | 0.003 | -0.006 to 0.012 | 0.506 |
| Hepatic DO_2_ (ml/kg/min) | 0.191 | -0.022 to 0.404 | 0.079 |
| Hepatic VO_2_ (ml/kg/min) | -0.080 | -0.633 to 0.474 | 0.778 |
| Hepatic O_2_ER | -2.752 | -5.033 to -0.470 | 0.018 |
| Hepatic lactate uptake (µmol/kg/min) | 0.113 | 0.016 to 0.221 | 0.023 |
| Hepatosplanchnic lactate uptake (µmol/kg/min) | -0.092 | -0.182 to -0.002 | 0.044 |
| Lactate hepatic vein (mmol/L) | 0.065 | -0.108 to 0.238 | 0.461 |
| Lactate hepatic vein - hepatic artery (mmol/L) | -2.406 | -4.775 to -0.038 | 0.046 |
| Lactate hepatic vein - portal vein (mmol/L) | 2.324 | -0.015 to 4.663 | 0.052 |

**β** = unstandardized coefficient, CI = confidence interval, MAP = mean arterial blood pressure, CVP = central venous pressure, pCO_2_ = carbon dioxide partial pressure, pO_2_ = oxygen partial pressure, total hepatic blood flow = hepatic arterial blood flow + portal vein blood flow, total hepatosplanchnic blood flow = celiac trunk blood flow + superior mesenteric artery blood flow, DO_2_ = oxygen delivery, VO_2_ = oxygen consumption, O2ER = oxygen extraction, hepatic lactate uptake = hepatic lactate influx − hepatic lactate efflux, hepatosplanchnic lactate uptake = (arterial lactate - hepatic venous lactate) * total hepatic blood flow.

**Table S6.** Correlation between renal venous-arterial CO_2_ to arterial-venous O_2_ content difference ratio (Cv-aCO_2_/Ca-vO_2_) and systemic and regional hemodynamics, kidney vein hemoglobin and blood gas analysis, and lactate uptake (n=170; including only systemic Cv-aCO_2_/Ca-vO_2_ >1.0).

| **Parameters** | **β** | **95%CI** | **p value** |
| --- | --- | --- | --- |
| MAP-CVP (mmHg) | -0.003 | -0.009 to 0.003 | 0.361 |
| Hemoglobin kidney vein (g/dl) | 0.014 | -0.055 to 0.083 | 0.694 |
| pH kidney vein | 1.089 | -8.770 to 10.948 | 0.829 |
| pCO_2_ kidney vein (mmHg) | 0.015 | -0.047 to 0.076 | 0.640 |
| pO_2_ kidney vein (mmHg) | 0.029 | 0.008 to 0.050 | 0.008 |
| Base excess kidney vein (mmol/L) | 0.075 | -0.077 to 0.227 | 0.334 |
| Renal artery blood flow (mL/kg/min) | 0.064 | -0.033 to 0.161 | 0.197 |
| Renal DO_2_ (ml/kg/min) | -0.764 | -1.718 to 0.190 | 0.117 |
| Renal VO_2_ (ml/kg/min) | 0.034 | -2.585 to 2.654 | 0.980 |
| Renal O_2_ER | 0.591 | -1.386 to 2.567 | 0.558 |
| Lactate kidney vein (mmol/L) | 0.192 | 0.019 to 0.365 | 0.030 |
| Kidney lactate uptake (µmol/kg/min) | 0.009 | -0.066 to 0.084 | 0.812 |
| Lactate gradient (mmol/L) | -0.431 | -0.778 to -0.083 | 0.015 |

**β** = unstandardized coefficient, CI = confidence interval, MAP = mean arterial blood pressure, CVP = central venous pressure, pCO_2_ = carbon dioxide partial pressure, pO_2_ = oxygen partial pressure, DO_2_ = oxygen delivery, VO_2_ = oxygen consumption, O_2_ER = oxygen extraction, lactate gradient = lactate kidney vein - lactate arterial.

**Table S7.** Correlation between spleen venous-arterial CO_2_ to arterial-venous O_2_ content difference ratio (Cv-aCO_2_/Ca-vO_2_) and systemic and regional hemodynamics, spleen vein hemoglobin and blood gas analysis, and lactate uptake (n=162; including only systemic Cv-aCO_2_/Ca-vO_2_ >1.0).

| **Parameters** | **β** | **95%CI** | **p value** |
| --- | --- | --- | --- |
| MAP-CVP (mmHg) | -0.006 | -0.015 to 0.003 | 0.194 |
| Hemoglobin spleen vein (g/dl) | 0.008 | -0.072 to 0.089 | 0.839 |
| pH spleen vein | 16.533 | -1.854 to 34.919 | 0.078 |
| pCO_2_ spleen vein (mmHg) | 0.128 | 0.021 to 0.236 | 0.019 |
| pO_2_ spleen vein (mmHg) | 0.005 | -0.008 to 0.017 | 0.490 |
| Base excess spleen vein (mmol/L) | -0.148 | -0.408 to 0.111 | 0.263 |
| Spleen artery blood flow (mL/kg/min) | 0.420 | -0.124 to 0.964 | 0.130 |
| Spleen DO_2_ (ml/kg/min) | -2.182 | -5.953 to 1.589 | 0.257 |
| Spleen VO_2_ (ml/kg/min) | -7.978 | -16.398 to 0.442 | 0.063 |
| Spleen O_2_ER | 0.055 | -1.587 to 1.697 | 0.948 |
| Lactate spleen vein (mmol/L) | -0.003 | -1.175 to 0.169 | 0.972 |
| Spleen lactate uptake (µmol/kg/min) | -0.142 | -0.667 to 0.382 | 0.595 |
| Lactate gradient (mmol/L) | -0.591 | -1.179 to -0.003 | 0.049 |

**β** = unstandardized coefficient, CI = confidence interval, MAP = mean arterial blood pressure, CVP = central venous pressure, pCO_2_ = carbon dioxide partial pressure, pO_2_ = oxygen partial pressure, DO_2_ = oxygen delivery, VO_2_ = oxygen consumption, O_2_ER = oxygen extraction, lactate gradient = lactate spleen vein - lactate arterial.

**Table S8.** Correlation between gut venous-arterial CO_2_ to arterial-venous O_2_ content difference ratio (Cv-aCO_2_/Ca-vO_2_) and systemic and regional hemodynamics, mesenteric vein hemoglobin and blood gas analysis, and lactate uptake (n=166; including only systemic Cv-aCO_2_/Ca-vO_2_ >1.0).

| **Parameters** | **β** | **95%CI** | **p value** |
| --- | --- | --- | --- |
| MAP-CVP (mmHg) | -0.005 | -0.009 to 0.000 | 0.039 |
| Hemoglobin mesenteric vein (g/dl) | -0.029 | 0.123 to 0.066 | 0.553 |
| pH mesenteric vein | -5.670 | -18.001 to 6.662 | 0.368 |
| pCO_2_ mesenteric vein (mmHg) | -0.026 | -0.103 to 0.051 | 0.510 |
| pO_2_ mesenteric vein (mmHg) | -0.016 | -0.050 to 0.019 | 0.379 |
| Base excess mesenteric vein (mmol/L) | 0.140 | -0.047 to 0.328 | 0.142 |
| Mesenteric artery blood flow (mL/kg/min) | -0.012 | -0.067 to 0.044 | 0.677 |
| Gut DO_2_ (ml/kg/min) | -0.039 | -0.645 to 0.567 | 0.899 |
| Gut VO_2_ (ml/kg/min) | 0.824 | 0.207 to 1.442 | 0.009 |
| Gut O_2_ER | -3.624 | -6.247 to -1.001 | 0.007 |
| Lactate mesenteric vein (mmol/L) | 0.218 | 0.081 to 0.355 | 0.002 |
| Gut lactate uptake (µmol/kg/min) | 0.011 | -0.015 to 0.036 | 0.414 |
| Lactate gradient* (mmol/L) | -0.073 | -0.633 to 0.487 | 0.797 |

**β** = unstandardized coefficient, CI = confidence interval, MAP = mean arterial blood pressure, CVP = central venous pressure, pCO_2_ = carbon dioxide partial pressure, pO_2_ = oxygen partial pressure, DO_2_ = oxygen delivery, VO_2_ = oxygen consumption, O_2_ER = oxygen extraction, lactate gradient = lactate mesenteric vein - lactate arterial.

**Table S9.** Correlations between liver venous-arterial CO_2_ to arterial-venous O_2_ content difference ratio (Cv-aCO_2_/Ca-vO_2_) and systemic and regional hemodynamics, liver vein hemoglobin and blood gas analysis, and lactate uptake (n=171; including only systemic Cv-aCO_2_/Ca-vO_2_ >1.0).

| **Parameters** | **β** | **95%CI** | **p value** |
| --- | --- | --- | --- |
| MAP-CVP (mmHg) | -0.007 | -0.011 to -0.003 | 0.002 |
| Hemoglobin hepatic vein (g/dl) | -0.044 | -0.111 to 0.022 | 0.192 |
| pH hepatic vein | 11.246 | -2.334 to 24.826 | 0.105 |
| pCO_2_ hepatic vein (mmHg) | 0.093 | 0.007 to 0.179 | 0.035 |
| pO_2_ hepatic vein (mmHg) | -0.065 | -0.102 to -0.028 | 0.001 |
| Base excess hepatic vein (mmol/L) | -0.133 | -0.312 to 0.045 | 0.143 |
| Total hepatic blood flow (mL/kg/min) | -0.025 | -0.055 to 0.005 | 0.109 |
| Total hepatosplanchnic blood flow (mL/kg/min) | 0.007 | -0.008 to 0.021 | 0.363 |
| Hepatic DO_2_ (ml/kg/min) | 0.141 | -0.165 to 0.447 | 0.366 |
| Hepatic VO_2_ (ml/kg/min) | 0.150 | -0.643 to 0.943 | 0.711 |
| Hepatic O_2_ER | -4.404 | -7.126 to -1.682 | 0.002 |
| Hepatic lactate uptake (µmol/kg/min) | 0.100 | 0.007 to 0.192 | 0.035 |
| Hepatosplanchnic lactate uptake (µmol/kg/min) | -0.105 | -0.182 to -0.029 | 0.007 |
| Lactate hepatic vein (mmol/L) | 0.111 | -0.046 to 0.268 | 0.167 |
| Lactate hepatic vein - hepatic artery (mmol/L) | -3.047 | -5.236 to -0.859 | 0.006 |
| Lactate hepatic vein - portal vein (mmol/L) | 2.121 | -0.037 to 4.280 | 0.054 |

**β** = unstandardized coefficient, CI = confidence interval, MAP = mean arterial blood pressure, CVP = central venous pressure, pCO_2_ = carbon dioxide partial pressure, pO_2_ = oxygen partial pressure, total hepatic blood flow = hepatic arterial blood flow + portal vein blood flow, total hepatosplanchnic blood flow = celiac trunk blood flow + superior mesenteric artery blood flow, DO_2_ = oxygen delivery, VO_2_ = oxygen consumption, O_2_ER = oxygen extraction, hepatic lactate uptake = hepatic lactate influx − hepatic lactate efflux, hepatosplanchnic lactate uptake = (arterial lactate - hepatic venous lactate) * total hepatic blood flow.

**Table S10.** Contributors to systemic venous-arterial CO_2_ to arterial-venous O_2_ content difference ratio (Cv-aCO_2_/Ca-vO_2_) (n=147; including only systemic Cv-aCO_2_/Ca-vO_2_ >1.0).

| **Parameters** | **β** | **95%CI** | **p value** |
| --- | --- | --- | --- |
| Renal Cv-aCO_2_/Ca-vO_2_ | 0.099 | 0.039 to 0.159 | 0.001 |
| Spleen Cv-aCO_2_/Ca-vO_2_ | 0.046 | -0.019 to 0.111 | 0.162 |
| Gut Cv-aCO_2_/Ca-vO_2_ | 0.120 | 0.015 to 0.225 | 0.025 |
| Liver Cv-aCO_2_/Ca-vO_2_ | -0.323 | -0.508 to -0.138 | 0.001 |
| Hepatosplanchnic Cv-aCO_2_/Ca-vO_2_ | 0.641 | 0.274 to 1.008 | 0.001 |
| MAP-CVP (mmHg) | -0.002 | -0.003 to 0.000 | 0.025 |
| pO_2_ kidney vein (mmHg) | 0.000 | -0.004 to 0.005 | 0.828 |
| Lactate kidney vein (mmol/L) | -0.101 | -0.200 to -0.003 | 0.043 |
| Renal lactate gradient (mmol/L) | 0.139 | 0.012 to 0.266 | 0.032 |
| pCO_2_ spleen vein (mmHg) | -0.009 | -0.016 to -0.003 | 0.002 |
| Spleen lactate gradient (mmol/L) | 0.104 | 0.004 to 0.203 | 0.041 |
| Gut VO_2_ (ml/kg/min) | -0.005 | -0.181 to 0.170 | 0.952 |
| Gut O_2_ER | 0.114 | -0.339 to 0.567 | 0.623 |
| Lactate mesenteric vein (mmol/L) | 0.089 | -0.022 to 0.199 | 0.115 |
| pCO_2_ hepatic vein (mmHg) | 0.010 | 0.002 to 0.018 | 0.014 |
| pO_2_ hepatic vein (mmHg) | -0.003 | -0.012 to 0.005 | 0.425 |
| Hepatic O_2_ER | -0.318 | -0.798 to 0.162 | 0.194 |
| Hepatic lactate uptake (µmol/kg/min) | -0.005 | -0.012 to 0.002 | 0.156 |
| Hepatosplanchnic lactate uptake (µmol/kg/min) | 0.005 | -0.002 to 0.013 | 0.167 |
| Lactate hepatic vein - hepatic artery (mmol/L) | -0.073 | -0.255 to 0.110 | 0.435 |

β = unstandardized coefficient, CI = confidence interval, Cv-aCO_2_/Ca-vO_2_ = venous-arterial CO_2_ to arterial-venous O_2_ content difference ratio, MAP = mean arterial blood pressure, CVP = central venous pressure, pO_2_ = oxygen partial pressure, Renal lactate gradient = lactate kidney vein - lactate arterial, pCO_2_ = carbon dioxide partial pressure, spleen lactate gradient = lactate spleen vein - lactate arterial, VO_2_ = oxygen consumption, O_2_ER = oxygen extraction, hepatic lactate uptake = hepatic lactate influx − hepatic lactate efflux, hepatosplanchnic lactate uptake = (arterial lactate - hepatic venous lactate) * total hepatic blood flow.

**Table S11.** Contributors to systemic venous-arterial CO_2_ to arterial-venous O_2_ content difference ratio (Cv-aCO_2_/Ca-vO_2_) after adjusting for experimental group (n=255).

| **Parameters** | **β** | **95%CI** | **p value** |
| --- | --- | --- | --- |
| Experimental group* |  |  | 0.292 |
| Renal Cv-aCO_2_/Ca-vO_2_ | 0.150 | 0.067 to 0.233 | <0.001 |
| Spleen Cv-aCO_2_/Ca-vO_2_ | 0.067 | 0.003 to 0.130 | 0.039 |
| Gut Cv-aCO_2_/Ca-vO_2_ | 0.120 | 0.031 to 0.209 | 0.008 |
| Liver Cv-aCO_2_/Ca-vO_2_ | -0.156 | -0.286 to -0.026 | 0.018 |
| Hepatosplanchnic Cv-aCO_2_/Ca-vO_2_ | 0.488 | 0.213 to 0.764 | 0.001 |
| MAP-CVP (mmHg) | 0.000 | -0.002 to 0.001 | 0.506 |
| Base excess kidney vein (mmol/L) | 0.000 | -0.013 to 0.013 | 0.981 |
| Renal O_2_ER | -0.007 | -0.251 to 0.236 | 0.953 |
| Lactate kidney vein (mmol/L) | -0.047 | -0.178 to 0.084 | 0.483 |
| Renal lactate gradient (mmol/L) | 0.106 | -0.036 to 0.249 | 0.142 |
| Spleen lactate gradient (mmol/L) | 0.034 | -0.058 to 0.127 | 0.469 |
| Gut O_2_ER | 0.112 | -0.161 to 0.384 | 0.422 |
| Lactate mesenteric vein (mmol/L) | 0.045 | -0.075 to 0.165 | 0.465 |
| pO_2_ hepatic vein (mmHg) | 0.000 | -0.006 to 0.007 | 0.899 |
| Total hepatic blood flow (mL/kg/min) | -0.002 | -0.009 to 0.006 | 0.695 |
| Hepatic O_2_ER | 0.103 | -0.170 to 0.377 | 0.458 |
| Hepatic lactate uptake (µmol/kg/min) | 0.000 | -0.008 to 0.008 | 0.943 |
| Hepatosplanchnic lactate uptake (µmol/kg/min) | 0.008 | -0.001 to 0.016 | 0.083 |
| Lactate hepatic vein - hepatic artery (µmol/L) | 0.091 | -0.126 to 0.308 | 0.411 |

* Experimental group: control group, endotoxin infusion, fecal peritonitis, cardiac tamponade and hypoxic hypoxia. ß = unstandardized coefficient, CI = confidence interval, Cv-aCO_2_ /Ca-vO_2_ = venous-arterial CO_2_ to arterial-venous O_2_ content difference ratio, MAP = mean arterial blood pressure, CVP = central venous pressure, O_2_ER = oxygen extraction, renal lactate gradient = lactate kidney vein - lactate arterial, spleen lactate gradient = lactate spleen vein - lactate arterial, pO_2_ = oxygen partial pressure, total hepatic blood flow = hepatic arterial blood flow + portal vein blood flow, hepatic lactate uptake = hepatic lactate influx − hepatic lactate efflux, hepatosplanchnic lactate uptake = (arterial lactate - hepatic venous lactate) * total hepatic blood flow.

**Table S12.** Contributors to systemic venous-arterial CO_2_ to arterial-venous O_2_ content difference ratio (Cv-aCO_2_/Ca-vO_2_) after adjusting for experimental group (n=147; including only systemic Cv-aCO_2_/Ca-vO_2_ >1.0).

| **Parameters** | **β** | **95%CI** | **p value** |
| --- | --- | --- | --- |
| Experimental group* |  |  | 0.449 |
| Renal Cv-aCO_2_/Ca-vO_2_ | 0.109 | 0.043 to 0.176 | 0.001 |
| Spleen Cv-aCO_2_/Ca-vO_2_ | 0.045 | -0.018 to 0.108 | 0.160 |
| Gut Cv-aCO_2_/Ca-vO_2_ | 0.125 | 0.028 to 0.222 | 0.011 |
| Liver Cv-aCO_2_/Ca-vO_2_ | -0.323 | -0.501 to -0.145 | <0.001 |
| Hepatosplanchnic Cv-aCO_2_/Ca-vO_2_ | 0.626 | 0.284 to 0.969 | 0.000 |
| MAP-CVP (mmHg) | -0.001 | -0.003 to 0.000 | 0.048 |
| pO_2_ kidney vein (mmHg) | 0.000 | -0.005 to 0.004 | 0.928 |
| Lactate kidney vein (mmol/L) | -0.109 | -0.212 to -0.005 | 0.039 |
| Renal lactate gradient (mmol/L) | 0.138 | 0.012 to 0.263 | 0.031 |
| pCO_2_ spleen vein (mmHg) | -0.009 | -0.015 to -0.003 | 0.006 |
| Spleen lactate gradient (mmol/L) | 0.114 | 0.012 to 0.215 | 0.029 |
| Gut VO_2_ (ml/kg/min) | -0.005 | -0.181 to 0.171 | 0.955 |
| Gut O_2_ER | 0.146 | -0.317 to 0.609 | 0.537 |
| Lactate mesenteric vein (mmol/L) | 0.099 | -0.026 to 0.224 | 0.120 |
| pCO_2_ hepatic vein (mmHg) | 0.010 | 0.002 to 0.018 | 0.014 |
| pO_2_ hepatic vein (mmHg) | -0.004 | -0.012 to 0.004 | 0.317 |
| Hepatic O_2_ER | -0.344 | -0.811 to 0.123 | 0.149 |
| Hepatic lactate uptake (µmol/kg/min) | -0.005 | -0.012 to 0.002 | 0.156 |
| Hepatosplanchnic lactate uptake (µmol/kg/min) | 0.004 | -0.003 to 0.012 | 0.273 |
| Lactate hepatic vein - hepatic artery (mmol/L) | -0.111 | -0.297 to 0.075 | 0.244 |

* Experimental group: control group, endotoxin infusion, fecal peritonitis, cardiac tamponade and hypoxic hypoxia. β = unstandardized coefficient, CI = confidence interval, Cv-aCO_2_/Ca-vO_2_ = venous-arterial CO_2_ to arterial-venous O_2_ content difference ratio, MAP = mean arterial blood pressure, CVP = central venous pressure, pO_2_ = oxygen partial pressure, Renal lactate gradient = lactate kidney vein - lactate arterial, pCO_2_ = carbon dioxide partial pressure, spleen lactate gradient = lactate spleen vein - lactate arterial, VO_2_ = oxygen consumption, O_2_ER = oxygen extraction, hepatic lactate uptake = hepatic lactate influx − hepatic lactate efflux, hepatosplanchnic lactate uptake = (arterial lactate - hepatic venous lactate) * total hepatic blood flow.

**Figure S1.** Linear regression and correlation between arterial lactate and base excess.


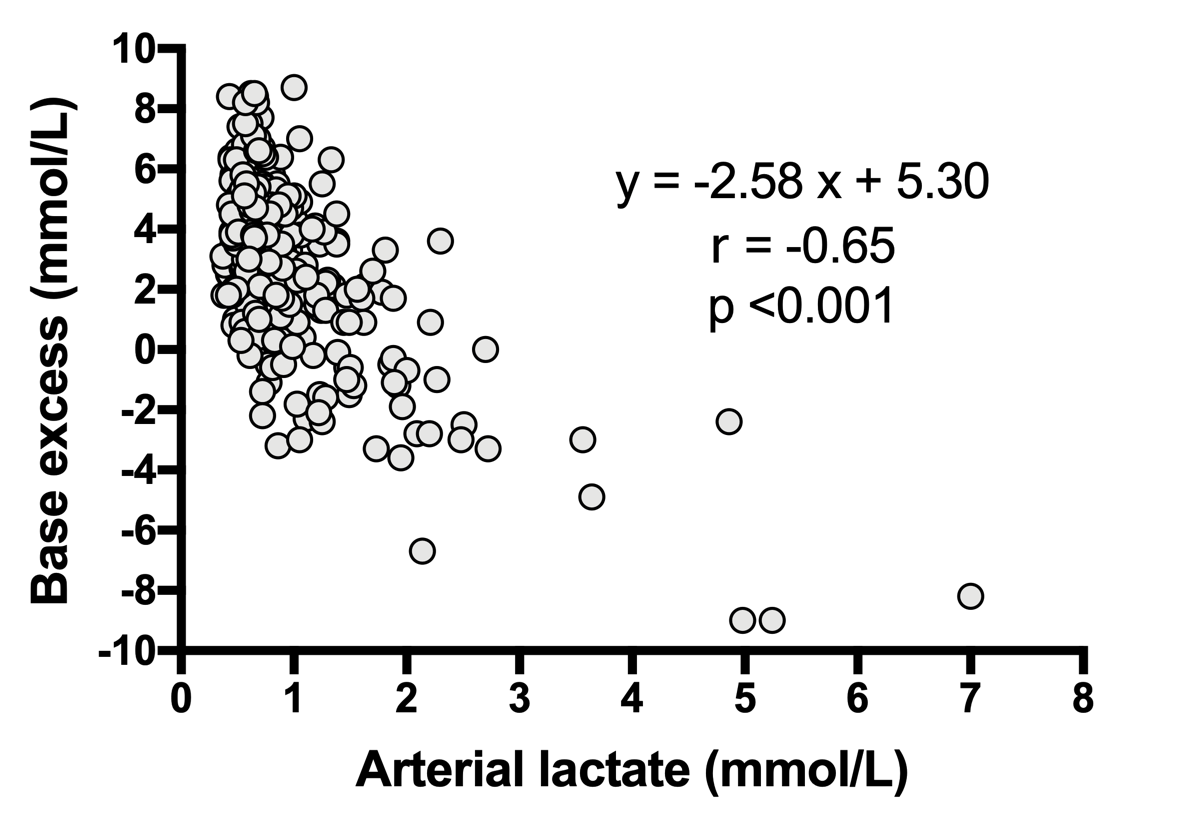


**Figure S2.** Boxplot of pooled hepatic, hepatosplanchnic, renal, mesenteric and spleen venous-arterial CO_2_ to arterial-venous O_2_ content difference ratio (Cv-aCO_2_/Ca-vO_2_).

**
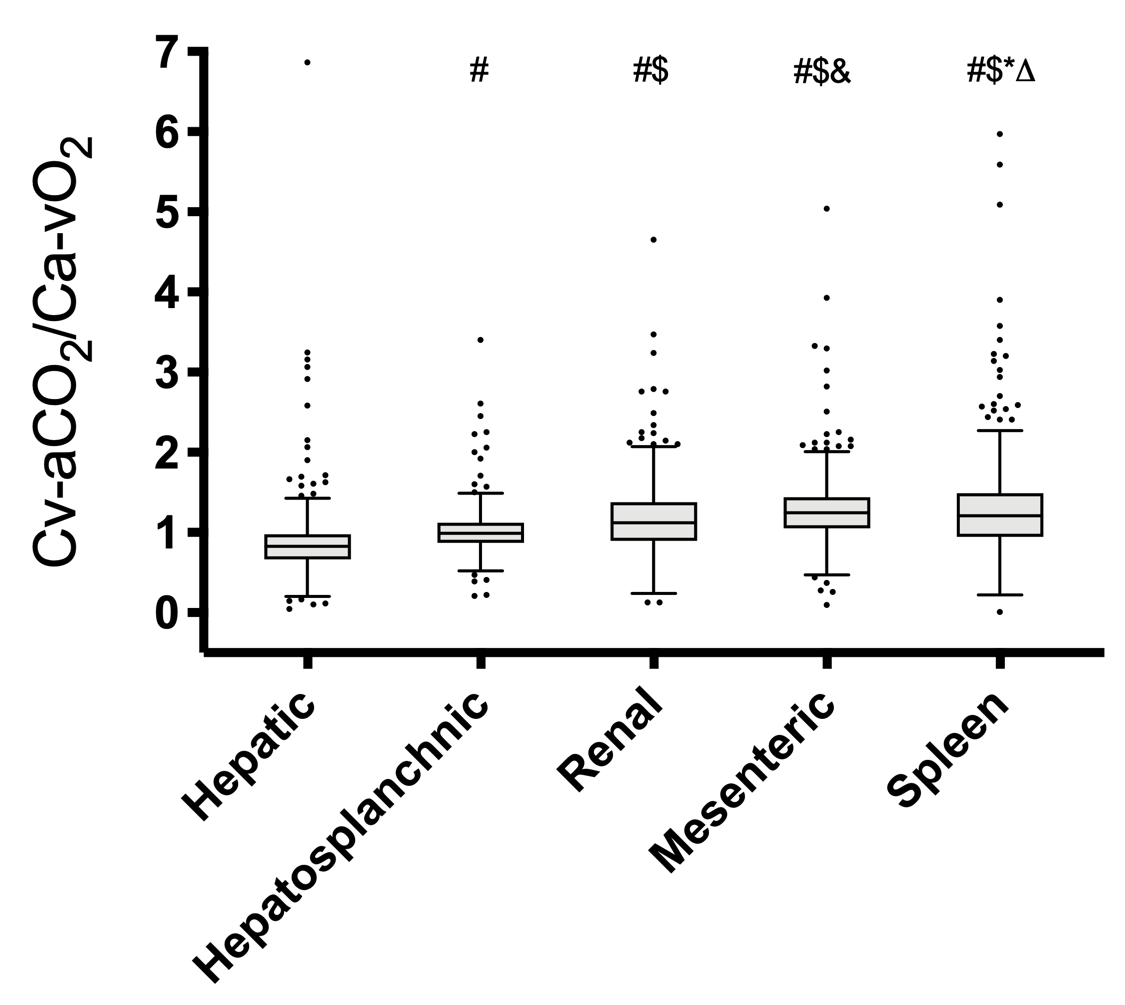
**

Cv-aCO_2_/Ca-vO_2_: venous-arterial CO_2_ to arterial-venous O_2_ content difference ratio. Pairwise comparisons were performed with Mann-Whitney U test after a significant (p <0.001) Kruskal-Wallis test. #: p <0.001 vs. Hepatic, $: p <0.001 vs. Hepatosplanchnic, &: p <0.001 vs. Renal, *: p =0.007 vs. Renal, ∆: p =0.314 vs. Mesenteric.

**Figure S3.** Systemic and regional CO_2_ to arterial-venous O_2_ content difference ratio (Cv-aCO_2_/Ca-vO_2_) accordingly to experimental models.


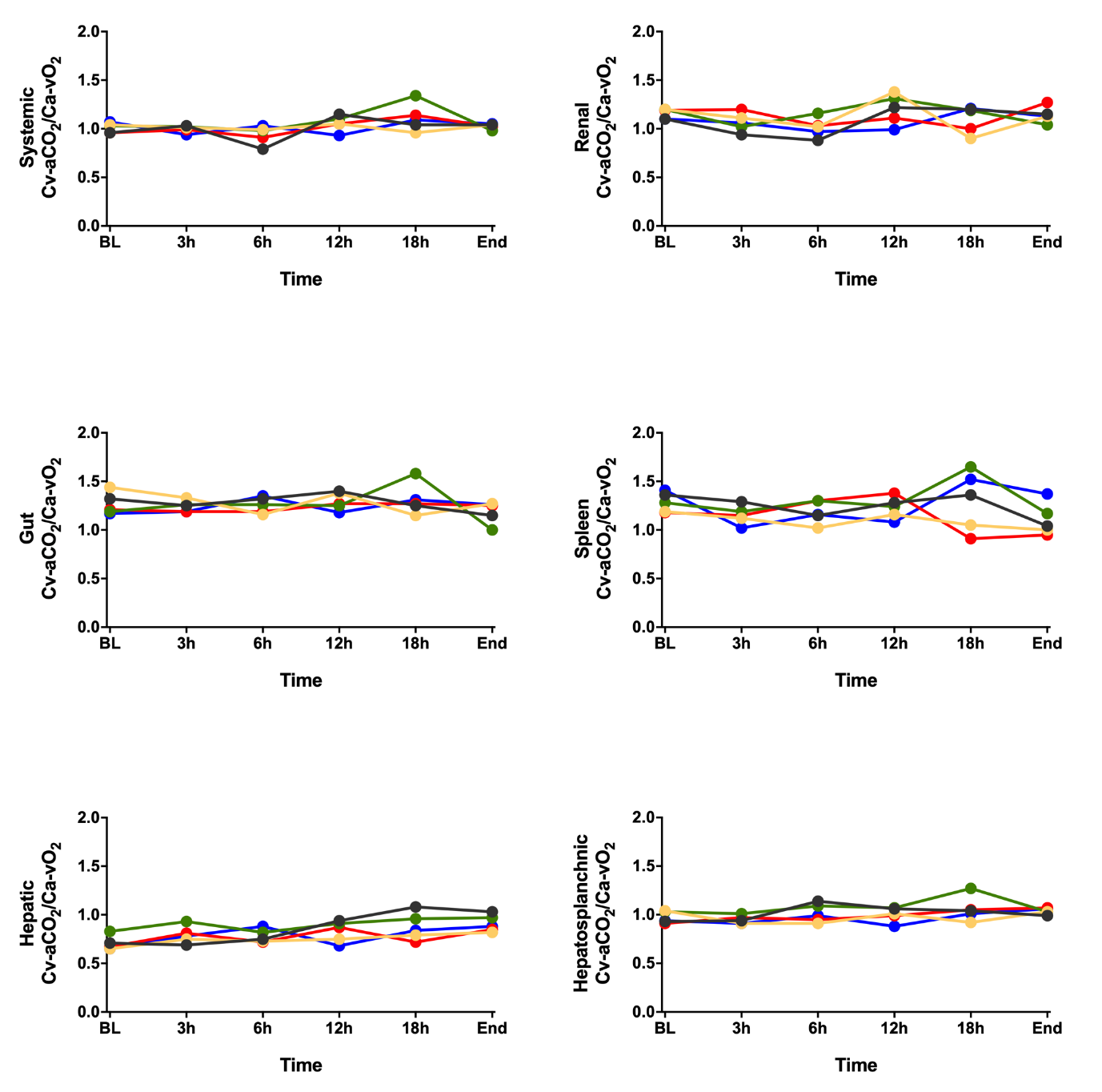


Values represent median. Blue lines: control group, black lines: hypoxic hypoxia, yellow lines: cardiac tamponade, green lines: fecal peritonitis, red line: endotoxin infusion. BL = baseline, End = end of experiment after 24 hours of randomization or before death, if earlier.
